# Hainan Longevity Model, Senile Degeneration, Cognitive Disability and Healthy Longevity

**DOI:** 10.1002/agm2.70054

**Published:** 2026-02-20

**Authors:** Shihui Fu, Jiacai Lin, Qianshuo Liu, Lianbo Gao, Zhirui Li, Feng Lin, Leixing Xie, Xiong Liu, Wenkai Xiao, Qiwei Zhu, Fan Wang, Jin Zheng, Yali Zhao, Xiaolei Chen, Yao Yao, Long Feng

**Affiliations:** ^1^ Department of Cardiology Hainan Hospital of Chinese PLA General Hospital, Hainan Geriatric Disease Clinical Medical Research Center, Hainan Branch of China Geriatric Disease Clinical Research Center Sanya China; ^2^ Department of Geriatric Cardiology Chinese PLA General Hospital Beijing China; ^3^ The Second School of Clinical Medicine Southern Medical University Guangzhou China; ^4^ Department of Neurology Hainan Hospital of Chinese PLA General Hospital Sanya China; ^5^ Department of Neurology the Fourth Affiliated Hospital of China Medical University Shenyang China; ^6^ Department of Orthopedics Hainan Hospital of Chinese PLA General Hospital Sanya China; ^7^ Medical Department Hainan Hospital of Chinese PLA General Hospital Sanya China; ^8^ Medical Care Center Hainan Hospital of Chinese PLA General Hospital Sanya China; ^9^ Department of Cardiology the Second Medical Center of Chinese PLA General Hospital Beijing China; ^10^ Central Laboratory, Hainan Hospital of Chinese PLA General Hospital Sanya China; ^11^ Department of Neurosurgery the First Medical Center, Chinese PLA General Hospital Beijing China; ^12^ Center for Healthy Aging Transdisciplinary Sciences, China Center for Health Development Studies Peking University Beijing China; ^13^ Department of Anesthesiology Hainan Hospital of Chinese PLA General Hospital Sanya China

**Keywords:** centenarians, cognitive disability, Hainan longevity model, healthy longevity, senile degeneration

## Abstract

The aging process is an inevitable and universal phenomenon characterized by a decline in function that all living organisms must undergo. The challenges posed by population aging, along with senile degeneration and cognitive disability, have emerged as pressing public health issues that require urgent attention on a global scale. The ability of older adults to maintain robust physiological function may confer resistance against the risk factors associated with senile degeneration and cognitive disability. Investigating centenarians who exemplify healthy longevity and their underlying mechanisms of functional resilience can aid in formulating preventive strategies aimed at achieving health, extending the lifespan, and identifying the pathways for promoting healthy longevity in the context of population aging. Hainan is the province in China where the largest number of centenarians and the highest density of centenarians are distributed. There are unique advantages to studying the centenarian population in Hainan. Hainan centenarians not only live in the natural environment with high oxygen concentration and dense greenery coverage but also inherit favorable genetic traits and adopt healthy lifestyles, including social participation, good education, stable emotion, balanced nutrition, moderate exercise, reasonable sleep, optimized senses, coordinated metabolism, improved immunity, prevention and treatment of chronic diseases, and joint treatment of comorbidities. These factors work together to form the Hainan longevity model (HLM), which is expected to play key roles in promoting healthy longevity and provide scientific evidence for achieving healthy longevity in the older populations. It will also contribute to the construction of a healthy human society and has important social value and practical significance.

## Graphical abstract

Investigating centenarians who exemplify healthy longevity can aid in formulating preventive strategies aimed at achieving health and extending lifespan in the context of population aging. Hainan longevity model (HLM) plays key roles in promoting healthy longevity and provides scientific evidence for achieving healthy longevity in the older population.

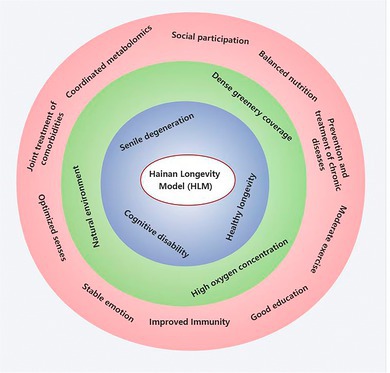

## 1 Introduction

Senile degeneration is an inevitable process of functional degeneration that all living things have to experience. There are currently 617 million (8.5%) older people aged 65 years and over in the global population. This is projected to increase to 1 billion (12%) by 2030 [1, 2]. Maintaining strong functioning in older adults may be resistant to risk factors for senile degeneration. Cognitive function is the core indicator of healthy longevity. Cognitive disability, defined as age‐related cognitive decline (ARCD), is a common phenomenon in the aging population. Age, sex, environmental factors, education level, living habits, physical ability, underlying diseases, and genetic susceptibility are common risk factors for senile degeneration and cognitive disability [3]. With the intensification of the global population aging process, the prevalence of cognitive disability has increased exponentially from 65.3 years old in 2015, and ARCD is diagnosed every 3 s in the world, which is expected to reach 152 million people in 2050 [4, 5]. There are about 10 million new cases of cognitive disability in older adults worldwide every year, and the number is expected to triple by 2050. The annual medical cost of cognitive disability is as high as $2 trillion. According to the results of the Fourth Sample Survey on the Living Conditions of China's Urban and Rural Elderly released by the Office on Aging in 2016, the number of disabled and semi‐disabled elderly in China is about 40.63 million, which is expected to reach 61.68 million in 2030 and 97.5 million in 2050. In addition, the number of cognitively disabled elderly in China reached 12 million in 2018, accounting for about 5% of the older population and 20% of the total number of cognitively disabled elderly in the world [4]. Population aging, senile degeneration, and cognitive disability have become a global public health problem that needs to be solved urgently. Data from the seventh national census revealed an important increase in the number of centenarians in China, from about 36,000 in 2010 to about 119,000 in 2020, making China the country with the largest number of centenarians in the world.

**FIGURE 1:**
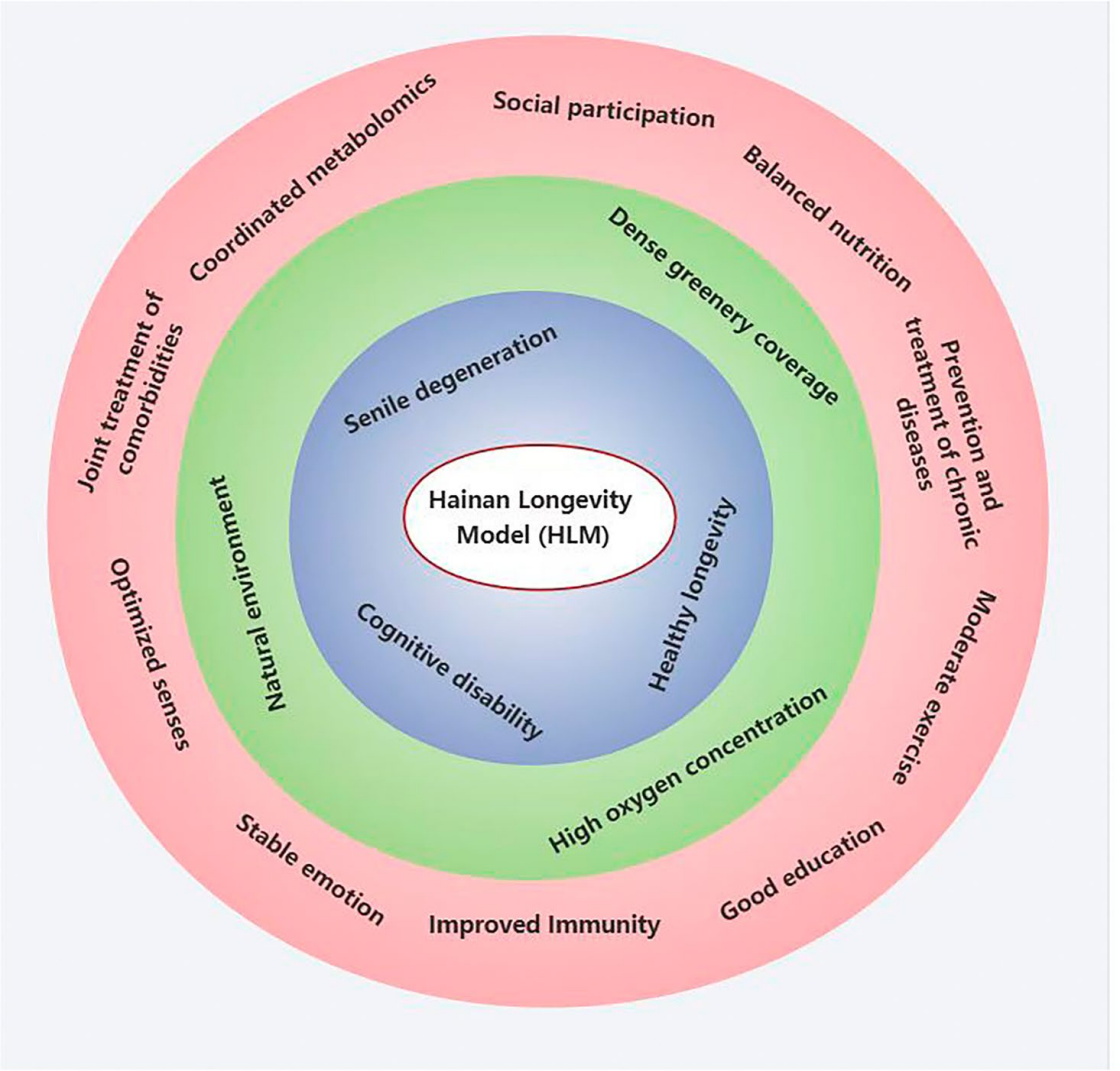
Hainan longevity model (HLM).

As the ultimate representative of the human life span, centenarians are of great significance to healthy aging by studying their longevity and cognition. Centenarians are a template population of healthy longevity, and their total number has increased year by year, which has become a new hotspot in epidemiological and geriatric research. Adherence to healthy lifestyles in later life plays key roles in effectively promoting healthy longevity [6]. Improved environment, socializing, diet, exercise, emotion, and sleep are positive factors for the effective prevention and control of senile degeneration and cognitive disability [7, 8, 9, 10, 11]. The Hainan centenarians' study also found that dietary nutrition, physical ability, and geriatric syndrome are related to life quality and healthy longevity [12]. Hainan is the province with the widest distribution and largest density of centenarians in China, known as the Longevity Island. Studying the etiology of senile degeneration and the mechanism of healthy longevity may help develop prevention and treatment strategies to maintain health and prolong lifespan [3]. What is particularly striking is that Hainan has far exceeded the standard of the United Nations Hometown of longevity, that is, 7.5 centenarians per 100,000 people, which is worthy of the name of the prestigious Longevity Island. The Hainan longevity model (HLM) is ecological characteristics and healthy lifestyles originated from long‐lived populations in Hainan. It comprehensively achieves healthy longevity through genetic interaction, environmental optimization, social participation, good education, stable emotion, balanced nutrition, moderate exercise, reasonable sleep, optimized senses, coordinated metabolism, improved immunity, prevention and treatment of chronic diseases, and joint treatment of comorbidities [13, 14, 15, 16, 17]. We propose HLM through a series of studies to explore environmental factors and common characteristics of Hainan centenarians (Figure 1). HLM not only benefits from Hainan's unique natural environment advantages but also covers healthy lifestyles such as light diet, positive emotion, and adequate sleep [18]. It is expected to reverse senile degeneration, improve cognitive disability, and promote healthy longevity, guiding the prevention and treatment of senile comorbidities and the development of geriatrics. Based on the real representatives of healthy longevity in Hainan centenarians, this concept is of great practical value and scientific significance for the construction of a healthy longevity human society.

## 2 Potential Mechanisms

Centenarians that survive to extremely old age are the result of interaction between genes, ecology, and lifestyle, and decreased mortality may be achieved by reducing risk exposure and inflammatory reaction and improving dietary nutrition and immune function. Extraordinary longevity of centenarians is a complex and heritable trait. The genetic component of healthy longevity spans generations and increases with survival beyond 90 years of age. Healthy longevity is associated with the presence of protective alleles or the absence of damaging alleles [19]. The healthy longevity of these individuals highlights a familial component and may result from the interaction of genetic factors and lipid metabolism [20]. Most centenarians eat and toilet by themselves. Of these, 80.3% were in good or fair health, 21.7% had good vision, 36.2% had good hearing, and 55.1% had their own teeth. Many centenarians work until they are 90 years or even 100 years. Centenarians maintain a regular lifestyle, with an adequate diet but low salt, low sugar, low fat, low calorie, low animal protein, high vitamin, and high fiber, and keep a calm temperament toward life and death [21]. In the aspect of their ecological environment, the natural environment is quiet and suitable, and the social environment is conducive to longevity. The neuroendocrine system also plays an important role in coordinating cell interaction, metabolism, growth, and senescence, contributing not only to quantitative longevity, but also to the prevention of age‐dependent and aging degenerative diseases [22].

Besides, the ability to survive to extremely old age has an important familial component. Recent studies have shown that genetic loci on chromosome 4 are associated with longevity, suggesting that at least one longevity gene may exist in humans [23]. Through deoxyribonucleic acid methylation changes at single CpG sites in our study on Hainan centenarians, we found that healthy longevity was related to deoxyribonucleic acid methylation changes at specific gene loci, and the combination of methylation profiles of HKR1, ROD1, and NLRC5 can be used as biomarkers for Hainan centenarians [24]. In addition, our study on Hainan centenarians also found that the single nucleotide polymorphism rs189037 in the Ataxia‐telangiectasia mutation gene was related to cardiac structure and healthy longevity, which increased left ventricular wall thickness and induced left ventricular eccentric hypertrophy, thereby shortening human life [25]. This study suggests that unmutated rs189037 is an indicator of healthy longevity, whereas mutated rs189037 may hinder human longevity. A study examining healthy longevity and oxidative stress showed that centenarians had lower levels of oxidized proteins, superoxide anion, coenzyme Q10, superoxide dismutase, and glutathione reductase, higher levels of vitamins A and E, reduced oxidative damage of lipids, and lower sensitivity to lipid peroxidation [26]. A review showed that ACE rs4340, ApoEε2/3/4, FOXO3A rs2802292, KLOTHO KL‐VS, and IL6 rs1800795 were associated with healthy longevity through meta‐analysis [27]. A study comparing ApoE allele/genotype frequencies in four centenarian cohorts suggests that ApoE may be a candidate to influence long‐lived populations. For individuals of different ethnic/geographic origins, the ApoEε4 allele may be detrimental to healthy longevity, while the ApoEε2 allele may be beneficial [28]. Moreover, not only ACE I/D gene single nucleotide polymorphisms are associated with healthy longevity, but the ACED allele and DD genotype may confer advantages that in turn lead to extraordinary longevity [29].

Neuropathological changes in the central nervous system are closely related to the cognitive disability of centenarians, and how to prevent or slow down neuropathological changes in the aging brain plays an important role in maintaining cognitive health [30]. Studies have found that about 15%–25% of centenarians have intact cognitive function, and about 90% of those with incomplete cognitive function at the age of 100 years delay clinical onset of cognitive disability until an average of 92 years of age [31]. Correlation studies in neuropathology and neuropsychology have also shown that some centenarians have no evidence of neurodegenerative disease and do not meet clinical criteria for dementia, even if biomarkers for Alzheimer's disease are present. These studies suggest that neuropathological changes in cognitive disability may be milder or delayed in centenarians. Epidemiological studies also show that dementia can be avoided even in centenarians or supercentenarians. Nearly half of cognitively disabled centenarians do not have adequate neuropathology to explain their cognitive symptoms, whereas about one‐third do not have cognitive disability, suggesting that some compensatory mechanisms, such as cognitive reserve or resilience, may play a role in helping centenarians escape cognitive disability [32]. Intervention strategies that improve overall status, maintain vascular health, and increase cognitive reserve may help preserve cognitive function into later life, thereby preventing cognitive disability and achieving healthy longevity.

The occurrence of cognitive disability is related to the mechanisms of nerve cell apoptosis, neurofibrillary tangles, amyloidosis, etc. It has also been pointed out that ARCD is associated with more complex and specific pathophysiological changes [33]. A study involving the donated brains of 40 centenarians found that cognitive performance measured before birth was associated with the distribution and quantity of ARCD‐related neuropathological markers (Aβ and Tau). After the autopsy, it was found that neuropathological signs and atherosclerotic changes associated with ARCD were present in most centenarians. Higher neuropathologic burden (neurofibrillary tangles, granulomatous degeneration, and amyloidosis) is associated with lower cognitive test performance and cognitive disability [34]. The neuropathological load of cognitively healthy centenarians is similar to that of patients with cognitive disability, suggesting that centenarians are resilient to the highest levels of cognitive disability neuropathology [35]. Transcriptomics analysis of samples from the hippocampus, a brain region related to cognition, found that several isoforms of metallothionein were highly expressed in centenarians, and they were mainly expressed in astrocytes [36]. Cognitive disability was associated with genetic stability (ApoE) [37], physiological indicators, dietary nutrition, physical ability, education level, social activities, cardiovascular diseases, and health self‐reporting [38]. Pathogenic mechanisms of cognitive disability may be related to different stress responses, such as heat stress, inflammatory stress, oxidative stress, metabolic stress, genotoxic stress, and protein homeostasis imbalance stress [39]. Brain aging is not an isolated degenerative process but is closely related to various organs and the external environment. Brain aging is related to the other seven systems such as the heart, lung, muscle, kidney, liver, immune, and metabolic systems, and cognitive disability is the result of multiple systems, organs, and factors [40, 41, 42]. At present, studies have not clearly pointed out the pathogenic mechanism of cognitive disability in centenarians, and neuropathological changes in centenarians' brains are similar to those in general dementia, but their pathological changes are milder and more delayed. Therefore, it is necessary to study the risk factors and protective factors of cognitive disability in centenarians to screen out scientific and healthy ecological models to delay and improve cognitive disability during the aging process and to provide the scientific basis for promoting brain health and guiding brain health care.

## 3 Natural Environment

Hainan is located in the southernmost part of China. With an area of about 35,400 km^2^, it is the second‐largest island in China. The terrain of the island is dominated by mountains and hills, and there are broad plains and sandy beaches along the coast. Its climate belongs to the tropical monsoon climate, warm and humid throughout the year, with an average annual temperature between 22°C and 26°C, warm winters, hot summers, and abundant rainfall. The island is very rich in natural resources (including rainforest, coral reef, marine life, etc.). High oxygen concentration and dense greenery coverage provide a comfortable high oxygen environment. This is because green space and oxygen environment are positively correlated with cognitive function and healthy longevity throughout the lifespan [43]. The brain needs more than one‐fifth of the oxygen in the body to function properly, and appropriately raising oxygen concentration and increasing oxygen uptake are beneficial for cognitive function and healthy longevity [44]. Reverse studies have also confirmed that low atmospheric oxygen partial pressure at high altitudes inevitably affects the brain, and short‐term, long‐term, or lifelong exposure to a hypoxic environment reduces autonomic spatial attention and cognitive processing function [45].

Looking ahead to the 21st century, the continued burning of fossil fuels leading to air pollution and global warming also poses further challenges to healthy longevity. Air pollution has also been shown to be a modifiable risk factor for cognitive disability and healthy longevity. Long‐term exposure to fine particulate matter, such as PM2.5, is associated with the development of aging and cognitive disability [46, 47, 48]. A study published in the Lancet Healthy Longevity found that PM2.5 has a causal relationship with cognitive disability in the elderly, and improving air quality is beneficial to their cognitive function [46]. Solid fuel use was associated with a risk of cognitive disability and recommended that clean fuel use be critical for cognitive function [47]. Exposure to household air pollution from solid fuel combustion contributes to poor sleep quality in older adults [48]. The adverse effects of air pollution on life expectancy are also evident [49]. A survey on the spatiotemporal changes of three air pollutants (PM10, SO2, and NOx) and three life indicators found that PM10, SO2, and NOx were negatively correlated with life expectancy in the older population [50]. Maintaining high‐quality indoor and outdoor air, avoiding indoor use of polluting energy, and promoting the use of clean energy have positive effects on preventing cognitive disability and promoting healthy longevity. At the same time, global warming will not only lead to increased heat waves for the elderly but also increase ozone levels and promote pathogen spread [51]. Previous studies have detected spatial heterogeneity in the association between regional comprehensive environmental and socioeconomic variables and longevity indicators. Physical and geographical conditions and altitude have an important impact on human longevity, while socioeconomic factors have a more important impact on human longevity.

## 4 Social Psychology

With the increase of age, there are fewer social activities and more marginal roles in the family, which may also have a certain impact on cognitive disability in centenarians. The higher the family function score, the higher the Mini‐Mental State Examination of the elderly. The prevalence of family dysfunction as high as 52.2% was associated with an increased risk of cognitive disability [52]. Therefore, a good relationship with family members plays an important role in healthy longevity, and providing a harmonious family atmosphere is essential for the cognitive function of centenarians. Studies have also suggested providing home and informal care for the oldest‐old and promoting medical care and regulation through health education [53]. Being single without close friends and low levels of social interaction are associated with an increased risk of cognitive disability among Chinese centenarians [54]. Strong inclusiveness and solidarity among neighbors in the village can alleviate the impact of mental stress and loneliness on the brain health of the elderly. Studies investigating the effects of social activities (travel, volunteering, and public speaking) on cognitive function in centenarians have found that participation in lifestyle plays an important role in senile degeneration and cognitive disability, and personality traits are moderators of the relationship between participation in lifestyle and individual mental state. The elderly with emotional stability, extroverted openness, and a strong sense of responsibility have higher mental state scores [55]. Studies have shown that sleep latency (biological), life satisfaction (psychological), and social intimacy are predictors of lifespan in the health model of centenarians [56]. Life satisfaction also affects how centenarians construct their subjective evaluation of health status, and life satisfaction is also directly related to realistic well‐being [57].

Positive emotion alleviates cognitive disability and promotes healthy longevity in centenarians [58]. An American study showed that centenarians have good mental health despite declining physical function and social resources, indicating that they have strong psychological adaptability and resilience to adapt to age‐related challenges [59]. Active participation in social and social activities is critical for maintaining cognitive function and for healthy longevity. Social distance generated by epidemic isolation had a negative impact on the cognitive function of centenarians during the COVID‐19 pandemic [60]. Positive life attitude and self‐rated health status are positively correlated with cognitive function and healthy longevity in centenarians [61]. A systematic review summarizing the subjective well‐being of centenarians also showed that personal health perception was associated with higher levels of life satisfaction, while fatigue was associated with lower levels of life satisfaction and poor prognosis [62].

Mental health of centenarians has contributed to psychological characteristics of healthy longevity. Social expectations are associated with depressive symptoms and mental health [63]. There were differences in social resources between centenarians and octogenarians and between centenarians with different living conditions [64]. Less contact with relatives and friends and greater use of psychotropic medications were associated with more frequent psychological distress, and it was noted that identifying risk and protective factors for psychological distress and life satisfaction provides an opportunity for intervention to maintain mental health [65]. Centenarians who actively participate in social activities and maintain a positive attitude, emotional stability, and extroverted openness have positive effects on the prevention and treatment of cognitive disability and the promotion of healthy longevity.

## 5 Dietary Nutrition

Malnutrition is very common among centenarians, with only 12.3% of them having normal nutrition [66]. The precondition of diet for longevity is that malnutrition should be avoided to prevent weakness and diseases caused by anemia, hypoproteinemia, and reduced bone or muscle mass. Appropriate nutritional interventions can optimize inflammatory and immune responses, thereby improving disease resistance and reducing mortality rate [67, 68, 69]. Hainan centenarians' study also found that malnutrition was positively correlated with cognitive disability [70]. Promoting dietary diversity provides a simple and clear way to identify and screen individuals at high risk of cognitive disability, which may delay and reduce cognitive disability in the elderly, especially in low‐ and middle‐income countries [71]. A high‐quality diet is related to cognitive function, so it is necessary to avoid single diet, fixed food, and isolated nutrients. It is recommended to eat a variety of foods containing multiple nutrients and to eat more fresh vegetables, fruits, and cereals. Garlic and fish can help delay the occurrence of cognitive disability [67]. Results from the dietary behavior analysis showed that 94.6% of centenarians eat regularly, 80.4% eat three meals a day, 53.4% eat to 80% full at each meal, 88.7% eat vegetables, and 70% drink one or two glasses of water every day. Centenarians are fed a staple diet based on rice, with disfavored eggs, legumes, poultry, and dairy products. The main sources of meat are red meat and seafood; no one likes fried food, and 19.3% of people like sweetness [72]. The preferred cooking method for Hainan centenarians was boiling (85.19%), and their energy intake was low (1040 kcal/days). The energy supply ratios of the three major heat‐producing nutrients were carbohydrate (52.67%), fat (32.82%), and protein (14.69%), respectively, and high‐quality protein accounted for 58.97% of total protein. The intake of vegetables and aquatic products reached the recommended intake, the intake of salt met the standard of low salt, and the intake of other vitamins and trace elements was low [73]. Vitamin D is a steroid hormone that exerts pleiotropic effects on a wide range of tissues and systems. Experience with centenarians shows that severe vitamin D deficiency does not rule out longevity, but that strategies to prevent vitamin D deficiency can help slow the process of frailty in old age [74]. Hainan centenarians' study also found that zinc and chromium content in hair was related to cognitive disability [75]. In addition, adequate concentrations of iron and selenium may promote healthy longevity in centenarians, whereas excessive concentrations of phosphorus and lead are harmful to health and lifespan [76].

Diet is an important part of living organisms. A study examines feasible nutritional strategies to delay aging and prevent diseases in centenarians through epidemiological survey, model organism, and clinical analysis, and highlights the need to avoid malnutrition and frailty. The longevity diet based on a multi‐pillar approach is adjusted according to individual age and health status to achieve healthy longevity [77, 78]. It is characterized by moderate carbohydrate content, low but adequate and mainly plant‐based protein intake, and fat consumption from plant sources, which provides about 30% of energy. Global Burden of Disease studies and meta‐analyzes provide evidence in support of the longevity diet, with a sustained shift from a typical Western diet to an optimal diet rich in whole grains, legumes, and nuts, and reduced red and processed meats [79]. In addition, the longevity advantage in Japan is largely believed to be related to a healthy traditional diet that is low in calories but rich in nutrients, especially in the form of antioxidants and flavonoid phytonutrients. The diet associated with reducing the risk of chronic diseases is similar to a healthy traditional diet, that is, high in vegetables and fruits with rich phytonutrients and antioxidants but low in sugar, salt, meat, refined grains, saturated fats, and full‐fat dairy products. The characteristics of these diets, such as low glycemic load, low saturated fat, and high antioxidants, may help reduce the risk of cardiovascular and cerebrovascular diseases, cancer, and other chronic diseases through a variety of mechanisms, including reduced oxidative stress [80]. Ideally, the longevity diet also involves fasting for 12–13 h per day, which has been shown to be safe, feasible, and effective in many studies [81].

## 6 Physical Ability

Improved education and social participation are also associated with cognitive function and healthy longevity. Muscle strength and social activities decrease with age, which affects the cognitive function of centenarians. Aging is a continuum of biological change processes characterized by gradual adaptation, which may be influenced by both genetic and physiological factors [82]. The decline in lung function and loss of muscle mass leads to a gradual decline in maximal oxygen uptake and maximal muscle strength over time, thereby decreasing physical ability. Tea and fish consumption, and good vision and hearing ability, were protective factors for physical ability. Low education level and vitamin D deficiency are risk factors for physical ability [83]. The prevalence of impaired physical ability was relatively high in centenarians. Most centenarians have reduced instrumental activities and find it difficult to maintain their self‐physical ability, and only 10% of them can achieve full marks in self‐physical ability maintenance [84]. Our study on Hainan centenarians also found that the physical ability of centenarians was related to lifespan and mortality [85]. Physical ability may improve with family, social, and medical care. Cognitive function is associated with life experience, social participation, education level, occupational exposure, and activity patterns, while active social participation and high education level are associated with slower cognitive disability in later life [86, 87, 88]. Educational attainment was associated with cognitive function not only through past lifestyle but also through current activities. Four direct predictors (i.e., education, current social participation, current activities, and past lifestyle) explained 35% of the variance in cognitive function [89]. In addition, improving the care of the elderly would also greatly improve their quality of life, especially in the activities of daily living [90]. Some studies have also found that cognitive function is positively correlated with grip strength and walking time [91]. Therefore, maintaining an active lifestyle and improving physical ability is beneficial to the prevention and treatment of cognitive disability and the promotion of healthy longevity [92].

Frailty has an important impact on dependent life, falls, disability, comorbidities, health, and mortality in older adults. One study found that frailty was associated with subsequent physical ability, cognitive function, disease status, and self‐rated health status. Frailty plays an important role in determining the subsequent health and mortality of centenarians [93]. Active participation in group activities and avoidance of personal frailty can enhance well‐being in many health domains throughout the lifespan, improve life quality, increase muscle strength, enhance cognitive function, and promote mental health, providing important health benefits to the older population and promoting healthy longevity [94]. The integration of lifestyle and genetic background can change the balance and resist frailty by activating appropriate defense mechanisms such as antioxidants in response to external and internal stresses, thus potentially fulfilling the promise of healthy longevity [95]. Age‐related frailty in centenarians includes progressively reduced lung function, maximal oxygen uptake, maximal muscle strength, and sarcopenia. Gas exchange across the lung during exercise is limited, but this limited oxygen transport is mitigated by improved muscle mechanical efficiency, which may play a crucial role in maintaining physical function and healthy longevity in this population [96]. Exercise has a positive effect on the physical function and healthy longevity of centenarians and other older people, and there is a close relationship between physical ability and healthy longevity [82]. Spanish centenarians further reduced the number of steps per minute and per day; that is, physical ability continued to decline until the end of human life [97]. Studies have found that reduced muscle mass is associated with long‐term all‐cause mortality in centenarians and is a predictor of mortality in centenarian women [98]. Low‐intensity physical ability, including brisk walking and jogging, has anti‐aging effects and helps protect against late‐life degeneration and cognitive disability. Walking and other low‐intensity physical abilities have a great contribution to the healthy longevity of the elderly. Integrating walking into daily life and encouraging walk‐based physical ability can be an effective strategy to prevent senile degeneration and promote healthy longevity [99]. A study of Italian centenarians showed calorie restriction of 17.5 h between meals and exercise throughout the lifespan, suggesting that plant‐based foods and physical ability are associated with healthy longevity [100]. Our study examined the association between daily life, activity patterns, cognitive disability, and healthy longevity in Hainan centenarians. Housework and newspaper reading were suggested to improve cognitive function and promote healthy longevity. In addition, according to the analysis of different constitutions, we also showed that Qi‐depression was closely related to cognitive disability in centenarians, and it is expected to prevent and control cognitive disability by regulating Qi‐depression [101].

## 7 Sleep Function

Sleep function and emotional problems have also attracted much attention in cognitive disability and healthy longevity. Studies have found that the cognitive function of centenarians is related to sleep quality, emotional health, and physical ability [102]. Improving physical ability and social resources contributes to the emotional health and cognitive function of centenarians. A study of Hainan centenarians showed that there was a correlation between sleep function and cognitive disability in centenarians, and there was a correlation between sleep time > 9 h at night and severe cognitive disability, so it is necessary to monitor and protect cognitive function related to sleep quality in the elderly [103]. Sleep duration > 8 h is also associated with worse cognition, memory, and execution [104]. Longer sleep latency and a lower percentage of sleep efficiency were associated with cognitive disability [105]. Among centenarians, there is a positive association between sleep quality and duration, death risk, and healthy longevity [106]. The elderly with poor and average sleep quality had 26% and 10% higher risks of death in the next 3 years, respectively. Poor sleep quality and longer sleep duration are also associated with worse health after 3 years [107]. Centenarians go to bed early at night, have no problem falling asleep, generally get up early in the morning, take a nap in the afternoon, and do not take medicine before going to bed. The quality, quantity, and habit of sleep have a great impact on healthy longevity [108]. Improving sleep quality and avoiding excessive sleep play a role in preventing cognitive disability and promoting healthy longevity [109].

## 8 Sensory Function

Age‐related hearing loss (ARHL) refers to the gradual age‐related loss of hearing, which is usually caused by irreversible degeneration of the inner ear or auditory nerve. About one‐third of people over 65 years old suffer from ARHL, and up to 75% of people over 80 years old. Managing hearing loss may reduce the risk of dementia. A study of older men found that patients with ARHL had a higher risk of ARCD than those without ARHL. Hainan Centenarians' study also found that there was a positive correlation between ARHL, ARCD, and depression [110]. For these deaf older adults, active wearing of hearing aids may help to improve cognitive disability, possibly because of social isolation and loneliness caused by hearing loss. The activation of the central auditory pathway is reduced, causing the compensatory activation of the cognitive control network [111]. In addition to hearing loss, centenarians often experience tooth loss. Some studies also reported that the average number of missing teeth in centenarians was 22. Complete dentures (65.5%) or partial dentures (21.8%) in at least one jaw were most common in centenarians. Cognitive disability, tooth loss, and the number of dentures are associated with impaired oral health‐related life quality [112]. It has also been reported that 83% of all centenarians are edentulous and wear removable complete maxillary and mandibular dental prostheses; 1% are edentulous without dental prostheses; 16% were not edentulous, with or without removable partial restorations (10% and 6%, respectively). These centenarians experience similar dry mouth, chewing difficulty, oral pain, and cognitive disability [113]. One study found that centenarians were less likely to have an edentulous jaw between the ages of 65 and 74 years. The number of teeth in the elderly affects life expectancy, and tooth loss is a predictor of healthy longevity [114].

## 9 Hormone Levels

Healthy longevity in centenarians was negatively correlated with abdominal obesity and positively correlated with sex hormones and bone turnover [115]. Overnutrition and abdominal obesity have an inverse relationship with sex hormones and bone turnover, and increased sex hormones and bone turnover may be representative of healthy longevity in centenarians. Avoiding overnutrition and abdominal obesity can increase sex hormones and bone turnover and promote healthy longevity in centenarians. Hormone levels are associated with physical ability and cognitive function in female centenarians [116]. Serum estradiol may be positively correlated with healthy longevity, while serum testosterone may be negatively correlated with healthy longevity [117]. Some studies have also found that centenarians have the key characteristics of fewer and delayed childbearing, suggesting that female fertility is negatively correlated with healthy longevity. For both men and women, centenarians are distinguished by later age at first and last childbearing. Less and later childbearing is an important factor in healthy longevity, and reduced and delayed childbearing in women may promote healthy longevity [118].

## 10 Immune Function

Immunity plays an important role in extreme old age, and centenarians rely on immunity to delay senile degeneration. Hainan centenarians' study found that the immune system with increased immunoglobulin E and decreased complement C3 and C4 is expected to prevent metabolic syndrome and abdominal obesity and can be used as therapeutic targets for promoting healthy longevity [119]. Hainan centenarians had less periumbilical fat, a weaker complement system, and a smaller heart diameter, while periumbilical fat, complement C3, and cardiac hypertrophy were inversely associated with healthy longevity [120]. Immunity does not appear to truly decline during aging, but the immune system undergoes remodeling, which is manifested in the residual function of memory cells, sometimes stronger, in contrast to the production and number of new antigen‐reactive naive T cells [121]. Single‐cell Ribonucleic Acid sequencing of peripheral blood from a random sample of centenarians was used to analyze the changes in the ratio of lymphocytes to bone marrow cells with aging, as well as the distribution of non‐cytotoxic and cytotoxic cells. An important change from the CD4^+^ T cell population to the B cell population was identified in centenarians, indicating a history of natural and environmental immunogen exposure. Transcriptional profiling identified cell‐type signatures that are unique to extreme longevity, including genes with age‐related changes (e.g., increased STK17A expression) and genes that are uniquely expressed in centenarians' peripheral blood monocytes (e.g., increased S100A4 expression) [122]. The study of immunophenotype, comorbidities, frailty, laboratory parameters, body mass index, and other clinical characteristics of Cuban centenarians found that the memory subset was dominant over the naive compartment in CD4^+^ and CD8^+^ T cells. Terminally differentiated CD8^+^CD28^−^ T cells were higher in frail centenarians than in prefrail centenarians, whereas CD8^+^CD57^+^ and CD8^+^EMRA T cells were higher in moderate‐to‐severe dependent individuals than in independent individuals [123].

## 11 Chronic Diseases

Hypertension is the most common condition associated with multiorgan comorbidities, disability, and death. The prevalence of hypertension in centenarians was 64.4%, with 58.9% of them under control. Hypertension and diabetes were risk factors for failure to live longer [124]. Clinical and functional status studies showed that the elderly who survived for 180 days had higher systolic blood pressure, diastolic blood pressure, mean arterial pressure, pulse pressure, Mini‐Mental State Examination, Barthel index, Lawton instrumental activity of daily living, albumin and calcium, and lower C‐reactive protein, cystatin C, and creatinine. Systolic blood pressure ≥ 140 mmHg, diastolic blood pressure ≥ 90 mmHg, mean arterial pressure ≥ 100 mmHg, and pulse pressure ≥ 40 mmHg were associated with higher 180‐day survival, indicating that mildly elevated blood pressure is a marker of healthy longevity in centenarians [125]. Mildly elevated blood pressure correlates with cardiac function and indicates adequate cardiac function. Blood pressure is the main factor affecting blood flow to the brain and other organs in patients with atherosclerosis, which is also the main feature of senile degeneration [126]. Cognitive function and healthy longevity are dependent on blood pressure [127]. The Hainan centenarians' study also found that the cognitive function of centenarians was positively correlated with diastolic blood pressure and negatively correlated with antibody expression, possibly because hypotension and stroke are the main risk factors for cognitive disability [128]. Lower diastolic blood pressure and orthostatic hypotension in the older population have been shown to be strongly associated with an increased risk of cognitive disability. Decreased diastolic blood pressure restricts blood supply to the brain, resulting in reduced cerebral hemoperfusion and increased cognitive disability. Decreased blood pressure may be associated with small cerebral vascular diseases (lacunar infarction, leukoaraiosis, subcortical white matter lesion, or microbleeds) or large cerebral vascular diseases (stroke) [129, 130, 131, 132]. Lower systolic blood pressure and narrow pulse pressure are associated with cognitive disability, while higher systolic blood pressure and wide pulse pressure are associated with better functioning in centenarians [133]. Heart rate variability is a predictor of mortality in centenarians and plays an important role in healthy longevity [134]. A household survey showed a similar prevalence of obesity, hypertension, and dyslipidemia between offspring and offspring spouses of centenarians, whereas centenarians had a lower prevalence of obesity and a higher prevalence of hypertension. Lipids, waist circumference, body mass index, and diastolic blood pressure were lower, but systolic blood pressure was higher in centenarians [135]. There was an association between body mass index and cognitive function, with the least cognitive disability at body mass index 20 kg/m^2^ [136]. Fasting blood glucose and insulin affect cognitive function and healthy longevity in centenarians [137].

Chronic kidney disease may affect cognitive function and healthy longevity in the elderly. Hyperuricemia is also associated with cognitive disability. One study found that higher uric acid in centenarians was associated with a lower risk of cognitive disability [138]. Metabolic syndrome had an important relationship with renal function in centenarians. The changed prevalence of metabolic syndrome and risk factors explains the possible causes of healthy longevity, and the interaction between age, metabolic syndrome, risk factors, and renal function is the mechanism of healthy longevity [139]. Centenarians had normal hemoglobin and a mildly reduced glomerular filtration rate and did not require erythropoietic support. The inverse correlation between hemoglobin and two renal biomarkers (neutrophil gelatinase‐associated lipocalin and erythropoietin) confirmed a critical role of renal function in erythropoiesis at extreme longevity. Sex differences emerged with lower values for hemoglobin and glomerular filtration rates in women than in men [140]. As a good healthy longevity model, centenarian offspring are also more likely to live longer. Studies have shown improved blood lipids, renal function, and especially diastolic but not systolic blood pressure in centenarians and their offspring, suggesting that these factors may play an important role in family longevity and healthy longevity [141].

## 12 Biological Markers

Biomarker profiling of long‐lived individuals and their short‐lived peers can improve our understanding of healthy longevity. A biometric‐based follow‐up study over 35 years showed higher levels of total cholesterol and iron and lower levels of glucose, creatinine, uric acid, aspartate aminotransferase, γ‐glutamyltransferase, alkaline phosphatase, lactate dehydrogenase, and total iron binding ability in centenarians [142]. Leukocyte telomere length, dehydroepiandrosterone sulfate, and adiponectin are associated with nutrition and performance in centenarians. There were important correlations between performance, albumin, adiponectin, dehydroepiandrosterone sulfate, and leukocyte telomere length. Short leukocyte telomere length is associated with poor performance and cancer development. Adiponectin or dehydroepiandrosterone sulfate is associated with obesity development and nutritional status [143].

## 13 Metabolomic Analysis

Metabolomics is a new technology to study the functional state of organisms. Effects of metabolites and their changes on biological function are explored through the analysis of small molecule metabolites in organisms [144]. Circulating metabolites may be associated with lifestyle and senile degeneration, opening new avenues for the prevention of cognitive disability and healthy longevity [145]. The method of metabolomics to detect circulating metabolites can reflect the interaction of genetic and environmental factors and metabolic changes of brain or cerebrospinal fluid, with important application value for exploring the pathophysiology and identifying the biomarkers of cognitive disability and healthy longevity. Analysis of circulating metabolites in patients with cognitive disability showed that sphingomyelin and ether‐containing phosphatidylcholine were changed in the preclinical stage, while acylcarnitine and several amines (including the branched‐chain amino acid valine and alpha‐aminoadipic acid) were changed in the symptomatic stage [146]. Another study compared the targeted metabolites between postmortem blood and brain samples of patients with cognitive disability and found that more decanoyl carnitine, heptanoylcarnitine, and tecodienylcarnitine can predict the risk of cognitive disability [147]. Glutamine and O‐acetyl‐glycoprotein are associated with an increased risk of cognitive disability, whereas glutamate, tyrosine, acetate, glycine, and phenylalanine are inversely associated with an increased risk of cognitive disability [148].

Hainan centenarians' study found that characteristic metabolites associated with healthy longevity in centenarians mainly included phosphatidylserine, lysine phosphatidylethanolamine, phosphatidylcholine, phosphatidylinositol, bile acids, and amino acids. Increased acetic acid, propionic acid, butyric acid, valeric acid, and total short‐chain fatty acids were positively correlated with dietary fiber intake in centenarians. Age concomitant and dietary remodeling of phospholipids, amino acids, and short‐chain fatty acids express unique metabolic signatures associated with extreme longevity [149]. Metabolic remodeling reduces local inflammation, improves cognitive function, promotes healthy lifespan, enhances antioxidant ability, and shapes healthy longevity. A metabolomic and lipidomic analysis of centenarians revealed that the characteristic metabolic phenotypes of centenarians differ in terms of amino acids and lipids. Shotgun lipidomics showed the changed lipid synthesis in centenarians, and sphingolipids were biomarkers and bioregulators of healthy longevity [150]. Centenarians have better antioxidant ability and developed remodeling processes of membrane lipids in a healthy longevity phenotype.

## 14 Gut Microbiota

Resident bacteria in the gastrointestinal tract produce most neurotransmitters in the human brain, thereby affecting the nervous system and cognitive function [151, 152]. Gut microbiota comprises a complex microbial community that lives in the human gastrointestinal ecosystem. Its changes not only affect gastrointestinal diseases but also affect healthy longevity [153]. In recent years, with the proposal of the concept of the gut‐brain axis, relevant studies have confirmed that intestinal flora plays an important role in the regulation of brain function, and its role in the regulation of cognitive function has been gradually recognized, which has become an important potential method to delay cognitive disability [154]. Dysbiosis leads to an increased permeability of the gut and blood–brain barrier, which mediates or affects the occurrence of senile degeneration and cognitive disability [153]. Older people have reduced diversity and stability of gut microbiota. The content of Bacteroidetes is relatively rich, while that of Firmicutes is relatively low. As a healthy longevity population, centenarians have their own composition of gut microbiota, with Bacteroidetes and Proteobacteria as the main phylum [155]. One study confirmed that 27‐hydroxycholesterol treatment aggravated pathophysiological changes in cognitive disability, particularly gut microbiota dysregulation and gut barrier dysfunction, suggesting that their neurotoxic effects on cognitive function are promising as potential therapeutic targets [156]. The composition of gut microbiota also changes with aging and affects physiological function [157, 158]. Gut microbiota also secretes large amounts of amyloid and lipopolysaccharide, which help regulate signaling pathways associated with cognitive disability and produce proinflammatory cytokines involved in its pathogenesis. Studies have also suggested that the interaction between gut microbiota and microglia plays an important role in cognitive function and neuroinflammation [159]. Hainan centenarians' study found that Butyrate‐producing Bacteria decreased and *Escherichia coli* , Bacillus Suis, and Methanobacterium Smith increased in centenarians. These species showed a continuous trend of change with age [160]. Centenarians exhibit youth‐related features in the gut microbiota, including increased species evenness, consumption of pathogenic bacteria, and enrichment of beneficial Bacteroides species [161].

Healthy longevity may be the result of gut microbiota adapting to endogenous and environmental changes, continually reestablishing a reciprocal relationship with the host. Studies of gut microbiota in centenarians may provide insights into whether and how it contributes to healthy longevity [162]. Gut microbiota has emerged as a potential factor favoring healthy longevity and has been proposed as a determinant of healthy longevity [163]. Maintaining host‐microbial homeostasis improves intestinal permeability, counteracts inflammatory reactions, and prevents potential declines in bone, muscle, and cognitive function. *Bacteroides fragilis* , which is enriched in centenarians, may mediate a critical balance between health and disease by upregulating the expression of the anti‐inflammatory factor IL‐10, thereby promoting successful aging. Bifidobacterium pseudochain may be a particularly beneficial bacterium for improving impaired nervous system and immune function [164]. The cumulative abundant and highly present commensal bacterial taxa include Ruminococcaceae, Lachnospiraceae, and Bacteroidaceae, which are core microbiota and decrease with age, but these characteristics are maintained with healthy longevity [155].

## 15 Conclusions

Senile degeneration is an inevitable process of functional degradation that all living organisms have to undergo. Population aging, senile degeneration, and cognitive disability have become a global public health problem that needs to be solved urgently. The maintenance of strong functioning in older adults may be resistant to risk factors for late‐life degeneration and cognitive disability. Understanding centenarians, a template population of healthy longevity, and their mechanisms behind functional resilience will help to formulate prevention and treatment strategies to achieve the goals of maintaining health, extending the lifespan, and identifying the pathways to promote healthy longevity against the background of population aging. Hainan is the province with the widest distribution and the largest density of centenarians in China, so it has unique advantages to study Hainan centenarians. Hainan centenarians not only live in the natural environment with high oxygen concentration and dense greenery coverage, but also inherit favorable genetic traits and adopt healthy lifestyles. It includes genetic interaction, environmental optimization, social participation, good education, stable emotion, balanced nutrition, moderate exercise, reasonable sleep, optimized senses, coordinated metabolism, improved immunity, prevention and treatment of chronic diseases, and joint treatment of comorbidities. These synergies constitute HLM, which is expected to promote healthy longevity in the older population, play key roles in promoting healthy longevity, and help the construction of a healthy human society, which has important social value and practical significance.

## Author Contributions

All authors contributed to the study design, data collection, and paper draft. All authors have read and approved the manuscript.

## Funding

This project was supported by Natural Science Foundation of Hainan Province (822MS198, 824MS171, 821QN389, 821MS117, 824MS173, 821MS115, 823MS161), Hainan Province Clinical Medical Center, Academician Yu Mengsun’s Workstation Innovation Platform Research Project (YSPTZX202406), Hainan Clinical Medical Research Center Project (LCYX202201, LCYX202303, LCYX202106), Health Care Soft Science Argumentation Research Project (24BJZ22), and Joint Program on Health Science & Technology Innovation of Hainan Province (WSJK2024MS176, WSJK2024QN080, WSJK2025ZD223).

## Conflicts of Interest

The authors declare no conflicts of interest.
